# HIV-1 gp120 envelope glycoprotein determinants for cytokine burst in human monocytes

**DOI:** 10.1371/journal.pone.0174550

**Published:** 2017-03-27

**Authors:** Benoît Levast, Lucie Barblu, Mathieu Coutu, Jérémie Prévost, Nathalie Brassard, Adam Peres, Camille Stegen, Joaquín Madrenas, Daniel E. Kaufmann, Andrés Finzi

**Affiliations:** 1 Department of Microbiology and Immunology, and Microbiome and Disease Tolerance Centre, McGill University, Montreal, Quebec, Canada; 2 Centre de Recherche du Centre Hospitalier Universitaire de Montréal, Montreal, Quebec, Canada; 3 Department of Microbiology, Infectiology and Immunology, Université de Montréal, Montreal, Quebec, Canada; 4 Los Angeles Biomedical Research Institute at Harbor-UCLA Medical Center, Torrance, California, United States of America; 5 Department of Medicine, Université de Montréal, Montreal, Quebec, Canada; Deutsches Primatenzentrum GmbH - Leibniz-Institut fur Primatenforschung, GERMANY

## Abstract

The first step of HIV infection involves the interaction of the gp120 envelope glycoprotein to its receptor CD4, mainly expressed on CD4^+^ T cells. Besides its role on HIV-1 entry, the gp120 has been shown to be involved in the production of IL-1, IL-6, CCL20 and other innate response cytokines by bystander, uninfected CD4^+^ T cells and monocytes. However, the gp120 determinants involved in these functions are not completely understood. Whether signalling leading to cytokine production is due to CD4 or other receptors is still unclear. Enhanced chemokine receptor binding and subsequent clustering receptors may lead to cytokine production. By using a comprehensive panel of gp120 mutants, here we show that CD4 binding is mandatory for cytokine outburst in monocytes. Our data suggest that targeting monocytes in HIV-infected patients might decrease systemic inflammation and the potential tissue injury associated with the production of inflammatory cytokines. Understanding how gp120 mediates a cytokine burst in monocytes might help develop new approaches to improve the chronic inflammation that persists in these patients despite effective suppression of viremia by antiretroviral therapy.

## 1. Introduction

After more than 30 years, the HIV pandemia is still a major health problem with near 37 million people infected and 2 million new infections each year [1]. Despite major efforts by scientists around the globe, there is still no effective vaccine to prevent HIV-1 acquisition. Current anti-retroviral treatment (ART) regimens are very effective in controlling viral replication and bringing plasma viral loads below detection limits. HIV infection leads to major perturbations on immune system regulatory mechanisms as CD4 T cells depletion, maturation and exhaustion of T cells and a constant immune activation are hallmarks of this infection. Previous studies have shown that chronic immune activation plays a critical role in HIV pathogenesis, contributing, besides direct infection, to progressive destruction of the CD4 T cell compartment and to the dysfunction of multiple immune cell subsets [2, 3]. HIV-induced inflammation is also described in the gastro-intestinal mucosa, leading to translocation of bacterial products [4, 5] which create an inflammatory loop that impairs epithelium integrity. Moreover, systemic inflammation and immune dysregulation persist in a significant fraction of the ART-suppressed individuals and is associated with non-AIDS defining clinical complications such as cardiovascular disease and cancer, as well as increased morbidity and mortality [2]. HIV infection is then associated with multifactorial pathogenic mechanisms, patients still present evidence of immune impairment such as expansion of myeloid derived suppressor. This inflammatory response occurs despite IL-10 production, a well-known anti-inflammatory cytokine. IL-10 is up-regulated in multiple cell types during HIV virus infection [6, 7] and blockade of IL-10 improves HIV-specific CD4 and CD8 T cells function, showing that this pathway contributes to virus-specific immune impairment.

It has been well established that soluble gp120 is a pro-inflammatory molecule [8–11]. Indeed, literature in the field reports production of TNFa and IL-8 in the genital tract that might lead to the recruitment of neutrophils. IL-6 is also induced by gp120 as well as the chemokines CCL2 and CCL4, leading again to immune cells recruitment [12]. Altogether, these reports suggest that the gp120 envelope glycoprotein induces a cytokine outburst during infection. Therefore, gp120-mediated cytokine induction may help HIV to better escape the immune system, replicate and disseminate. Consistent with this model, diverse amounts of soluble gp120 were shown to be present in plasma and tissue from HIV-1-infected individuals even under successful ART-treatment [13–15]. Accordingly, a link between the amount of soluble gp120 present in the plasma of infected individuals and their immune status has been suggested [14]. HIV-induced inflammation has also been reported in the brain [16, 17] where HIV-1 gp120 was reported to affect neurons and glial cells by producing oxidative stress [18–20] and contribute to brain disease in HIV-1-infected individuals [21].

A molecular understanding of the gp120 determinants involved in cytokine production by CD4^+^ T cells and other CD4-expressing immune cells is still missing. Since the gp120 evolved to efficiently bind the CD4 receptor with nanomolar affinity [22], we made the assumption that envelope protein gp120 binding to CD4 is required for the cytokine burst observed in monocytes. Here, we report an extensive structure-function analysis of the requirements for primary human cell activation by gp120 leading to pro-inflammatory cytokine production.

## 2. Material and methods

### 2.1. gp120 constructs and reagents

Mutations (D368R, R419D) were introduced into the previously-described codon-optimized pcDNA3.1-HIV-1_YU2_ gp120 [23] using the QuikChange II XL site-directed mutagenesis protocol (Stratagene, Cedar Creek, TX, USA). Deletions of the V1, V2, V3 and V5 region of the codon optimized pcDNA3.1-HIV-1_YU2_ expression construct were made by replacing the sequence encoding 124–198 from the V1/V2 loop with a sequence encoding a GG linker, the sequence encoding 302–323 from the V3 loop with a sequence encoding a GGSGSG linker and the V5 by replacing residues 460–465 by a GSG linker as previously described [23, 24]. The presence of the desired mutations was confirmed by Sanger DNA sequencing. All residues are numbered according to the prototypic HXBc2 sequence [25].

For gp120 or sCD4 production, FreeStyle 293F cells (Invitrogen, Carlsbad, Ca, USA) were grown in FreeStyle 293F medium (Invitrogen) to a density of 1 × 10^6^ cells/ml at 37°C with 8% CO_2_ with regular agitation (125 rpm). Cells were transfected with a pCDNA3.1 plasmid encoding codon-optimized His(6)-tagged wild-type (wt) or mutant HIV-1_YU2_ gp120 or sCD4 expression plasmid using the Expifectamine^™^ 293 Transfection Kit as directed by the manufacturer (Invitrogen). One week later, cells were pelleted and discarded. The supernatants were filtered (0.22-μm-pore-size filter) (ThermoFisher Waltham, MA, USA), and the gp120 glycoproteins or soluble CD4 (sCD4) were purified by nickel affinity columns according to the manufacturer's instructions (Invitrogen). Fractions containing gp120 were concentrated using Centriprep-30K (EMDMillipore Billerica,MA, USA) centrifugal filter units following the manufacturer instructions. Monomeric gp120 was then purified by FPLC as described [23]. Monomeric gp120 preparations were dialyzed against phosphate-buffered saline (PBS) and stored in aliquots at −80°C. To assess purity, recombinant proteins were loaded on SDS-PAGE gels and stained with Coomassie blue as described [23].

### 2.2. Cell isolation and culture

Human peripheral blood mononuclear cells (PBMCs) were isolated from venous blood of healthy HIV-negative volunteers by Ficoll-Hypaque density gradient centrifugation. Volunteers gave their written informed consent in compliance with the IRB from the University of Montreal Hospital (CHUM), called "Comité d’éthique de la recherche du CHUM” (English translation: CHUM Research Ethics Committee) and with the IRB from McGill University Health Centre, called the “Centre for Applied Ethics”;both ethics committees approved this study. PBMCs were cultured in RPMI 1640 supplemented with 10% fetal bovine serum (R10), penicillin-streptomycin, L-glutamine, non-essential amino acids and pyruvate. Peripheral blood neutrophils were isolated by red blood cell lysis of the pellet following Ficoll-Hypaque centrifugation. Monocytes were isolated from PBMCs following instructions of commercial isolation kit from Stemcell (EasySep).

PBMCs or monocytes were seeded in 96-well plates (200,000 cells per well in 100 μL of media) and stimulated (100 μL of stimulant) with gp120 constructs controlled with an unstimulated control (mock). For inhibition experiments using maraviroc, wortmanin or rapamycin, cells were incubated for 1 h at 37°C prior to stimulation, and using 0.1% DMSO as a control when appropriate. Cell-free supernatants were collected and stored at -20°C until analyzed for detection of cytokines by enzyme-linked immunosorbent assay (ELISA) following the instructions of the manufacturer (eBioscience). Stem cell Hetasep was used to separate whole blood (WB) leukocytes to red blood cells. WB cells were washed in R10 medium before stimulation and migration tests.

### 2.3. gp120 stimulation

Stimulation experiments for all cell types and cultures were done at a concentration of 200 ng/ml (3.53 nM) of gp120 (WT). The equivalent of 3.53nM was used for the gp120 constructs with different molecular mass. Cell cultures were performed with one million cells in 1 mL for 4h (RT-qPCR) or 18h (ELISA or FACS) at 37°C. After stimulation cells were washed and then processed in accordance to the specific technical protocols.

### 2.4. Antibodies and flow cytometry

PBMCs were stimulated with positive control (*S*. *aureus* peptidoglycan) or mock control or with the different gp120 constructs. Cells were washed in PBS containing 2% FBS and 2 mM EDTA, blocked with 10% normal human serum for 15 min at 4°C, and then stained for CD3, CD14, and CD19 (1 μL of each antibody in 100 μL of PBS containing 2% BSA and 2 mM EDTA) for 30 min at 4°C in the dark. Dead cells were excluded using Zombie Aqua Dye (BioLegend). For intracellular staining of IL-10 and TNF- α, Brefeldin A was added 6 hours post-stimulation and cells were cultured overnight. Then, cells were stained for surface markers before being fixed, permeabilized and stained for IL-10 and TNF-α. For intracellular cytokine staining of IL-1, a mix of Brefeldin A, PMA and ionomycin was added 12 hours post-stimulation with gp120 during 4 hours and cells were then fixed and permeabilized for staining. Events were collected on a FACSFortessa II (BD) and analyzed using FlowJo software (Tree Star Inc.).

### 2.5. Migration assay

Cell mobility was assessed using a 3μm membrane transwell culture well system (Corning) by seeding 2.10^5^ cells in the insert in 2% FBS completed RPMI and in presence of 3.53nM of recombinant gp120 proteins or controls. After 18h in culture conditions, media were harvested and cell counts were evaluated using beads (eBioscience) to normalize acquisition on a FACS Fortessa.

### 2.6. RT-qPCR gene expression

mRNA was extracted by column purification (RNeasy Plus Kit, Qiagen). Reverse transcription reaction was performed based on superscript III protocol (Invitrogen). qPCR two step reactions were then performed on a Cycler iQ. Data *Cq* were analyzed using genex macro [26]. The reference genes to normalize the data, RPL19 and HPRT, were selected using geNorm [26]. Primers were purchased from IDT and are listed in Table 1.

**Table 1:** Human gene RT-qPCR primers list.

Gene	Primer sequences	Amplicon size (pb)	Accession number
CCL2	F- TGCTCATAGCAGCCACCTTC R- TCTCCTTGGCCACAATGGTC	189	NM_002982
CCL3	F- CAACCAGTTCTCTGCATCAC R- CTGCTCGTCTCAAAGTAGTC	111	NM_002983
CCL4	F- TCCTCGCAACTTTGTGGTAG R- TCCAGGTCATACACGTACTC	141	NM_002984
CCL20	F- TGATGTCAGTGCTGCTACTC R- GATGTCACAGCCTTCATTGG	146	NM_004591
CXCL2	F- GCCCAAACCGAAGTCATAGC R- AGGAACAGCCACCAATAAGC	154	NM_002089
CXCL10	F- CTAGAACTGTACGCTGTACC R- TTGATGGCCTTCGATTCTGG	175	NM_001565
**HPRT**	F- ATTGTAATGACCAGTCAACAGGG R- GCATTGTTTTGCCAGTGTCAA	117	NM_000194
IL-1b	F- CTCGCCAGTGAAATGATGGCT R- GTCGGAGATTCGTAGCTGGAT	144	NM_000576
IL-2	F- GTCACAAACAGTGCACCTAC R- TCCTGGTGAGTTTGGGATTC	127	NM_000586
IL-6	F- GTGTGAAAGCAGCAAAGAGG R- TGTTCTGGAGGTACTCTAGG	164	NM_000600
IL-8	F- TTGGCAGCCTTCCTGATTTC R- ATTTCTGTGTTGGCGCAGTG	170	NM_000584
IL-10	F- ACTTTAAGGGTTACCTGGGTTGC R- TCACATGCGCCTTGATGTCTG	111	NM_000572
IL-18	F- GATGGCTGCTGAACCAGTAG R- GCCGATTTCCTTGGTCAATG	191	NM_001562
IL-23p19	F- ATGAGAAGCTGCTAGGATCG R- TTTGAAGCGGAGAAGGAGAC	194	NM_016584
**RPL19**	F- GGCTCGCCTCTAGTGTCCT R- GCGGGCCAAGGTGTTTTTC	185	NM_000981
TNF-a	F- GCTGCACTTTGGAGTGATCG R- GCTACAACATGGGCTACAGG	132	NM_000594

Primers were designed using Clone Manager software. F: Forward primer; R: Reverse primer. The size of the PCR product is indicated and expected after a two-steps cycle real-time qPCR. Reference genes used for normalization are indicated in bold character. Accession number referred to NCBI website collection.

### 2.7. gp120 immunoprecipitation

For pulse-labeling experiments, 3 × 10^5^ 293T cells were transfected with pcDNA3.1-HIV-1_YU2_ gp120 variants using a standard calcium phosphate method. One day after transfection, cells were labeled with 55 μCi/mL [^35^S]-methionine/cysteine ([^35^S] protein labeling mix (Perkin-Elmer, Waltham, MA, USA) in Dulbecco’s modified Eagle’s medium lacking methionine and cysteine and supplemented with 5% dialyzed fetal bovine serum. After 24 hours, cell supernatants were recovered. Precipitation of radiolabeled HIV-1 envelope glycoproteins from supernatant was performed with a mixture of sera from HIV-1-infected individuals. Alternatively, radiolabeled gp120 envelope glycoprotein in the supernatant was precipitated with various amounts of anti-gp120 monoclonal antibodies or the recombinant CD4-Ig protein for 1 h at 37°C in the presence of 50 μl of 10% Protein A-Sepharose beads (GE Healthcare). The beads were then washed twice with RIPA buffer [140 nM NaCl, 8 mM Na2HPO4, 2 mM NaH2PO4, 1% NP40, 0.05% sodium dodecyl sulfate (SDS)]. Laemmli buffer without β-mercapto-ethanol was added to the beads before heating them at 100°C for 5 min and loaded on SDS-PAGE polyacrylamide gels.

To assess CCR5-binding ability, normalized amounts of radiolabeled gp120 envelope glycoproteins from transfected 293T cell supernatants were incubated in the presence or absence of sCD4 (10 μg/ml) for 1 hour at 37°C. The gp120-sCD4 mixtures were then incubated with 5 X 10^6^ Cf2Th-CCR5 cells for 2 hours at 37°C. Cells were then washed twice with PBS prior to lysis in RIPA buffer. The bound gp120 glycoproteins in the cell lysates were immunoprecipitated with a mixture of sera from HIV-1-infected individuals. Laemmli buffer with β-mercapto-ethanol was added to the beads before heating them at 100°C for 5 min and loaded on SDS-PAGE polyacrylamide gels. All precipitated proteins were analyzed on SDS-PAGE polyacrylamide gels. Gels were dried with a Model 583 gel dryer (Bio-Rad) and exposed in a storage phosphor cassette. Densitometry data was acquired with a Typhoon Trio Variable Mode Imager (Amersham Biosciences) storage phosphor acquisition mode. Results were analyzed using Image Quant 5.2 software (Molecular Dynamics).

## 3. Results

### 3.1. Low levels of gp120 induce a cytokine burst in human monocytes

**Fig 1:**
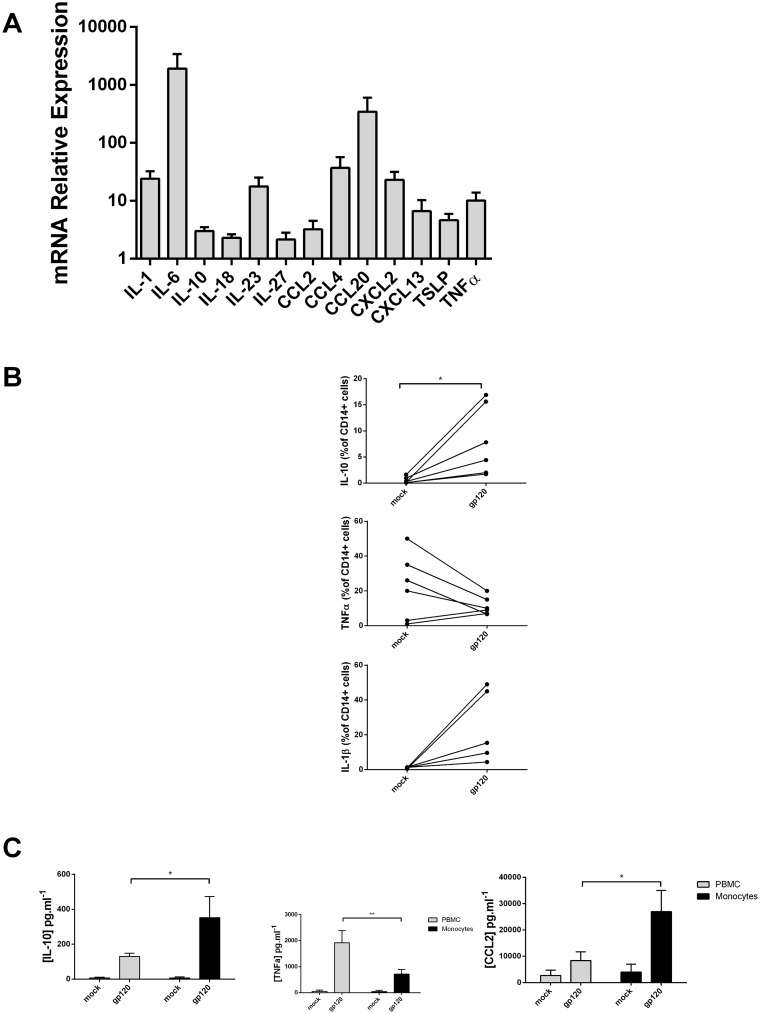
gp120 induces a cytokine burst in PBMC and monocytes. A) mRNA expression of cytokines done by RT-qPCR, level of expression after stimulation with gp120 is relative to the mock control set at 1 for each cytokine. B) Flow cytometry analysis of PBMC CD14+ population, expression of IL-10, TNF-α and IL1-ß are detected by intra-cellular staining (left panels). Data are represented in paired samples and statistic analysis performed with paired t-test (* p<0.05). C) PBMC (grey bars) and monocytes (black bars) supernatant analysis by ELISA detection of IL-10, TNF-α and CCL2 after stimulation with gp120. Statistic analysis performed with t-tests present the difference between the two cell type (* p<0.05 and ** p<0.01).

Whether gp120 signals through different receptors is an important question that remains unclear. To start addressing this question, we characterized the cytokine profile produced by PBMC after stimulation with levels of recombinant gp120 that have been reported *in vivo* or *in vitro* [13, 14, 27, 28]. Using RT-qPCR on PBMC (Fig 1A), we identified 13 different cytokines significantly over-expressed upon gp120 stimulation. These included interleukins -1, -6, -10, -18, -23, -27, CCL2, 4, 20, CXCL2, 13, TSLP and TNFα.

It is important to note that 200 ng of gp120 per million cells (per milliliter), a level recently reported to correspond to the amount of gp120 present at the surface of HIV-1-infected cells [27] and bystander uninfected CD4+ T cells [28], induced significant cytokine production. Of note, dose response experiments indicated that the concentration of gp120 used in this study (200 ng/1x10^6^/mL) does not result in maximal cytokine production (data not shown). Thus, indicating that our system is not saturated and suggesting that this cytokine response to gp120 might be representative of *in vivo* responses [13, 15]. Some of the cytokines upregulated upon gp120 exposure such as IL-1, IL-6 and CCL20 have important pro-inflammatory functions. The induction of IL-6 was particularly pronounced, (1000 fold increase, Fig 1A). One cytokine in particular, IL-10, has anti-inflammatory properties while CXCL13 is a B cell chemoattractant and Thymic Stromal Lymphopoietin (TSLP) is a regulator of myeloid and T cell functions.

Next, we studied at the protein level the cell populations producing the cytokines induced by gp120 stimulation. We used polychromatic flow cytometry and intracellular staining on gp120-stimulated cells and unstimulated controls (mock, Fig 1B). Interestingly, of all the PBMCs populations, only CD14^+^ monocytes appeared to respond to the gp120-stimulation by expressing IL-10 and/or TNF. CD4^+^, CD8^+^ and CD19^+^ lymphocytes did not respond in our ICS experiment (data not shown). We detected a mean of 8% of IL-10 producing monocytes. We observed variable trends in TNF-α production with induction or reduction of TNF-α upon gp120 stimulation (Fig 1B). Analysis of IL-1 production was more challenging because of the loss of CD14 expression after PMA/ionomycin activation. Indeed, activation of monocytes can lead to cell receptor downregulation [29]. Nevertheless, a positive staining for IL-1 was detected after stimulation of cells with gp120 (Fig 1B, third panel). PMA/ionomycin activation of cells revealed very few IL-1 positive mock-control cells versus a mean of 10.7% in the presence of gp120.

To corroborate these findings, we next analyzed secreted cytokines from cultured cells. We found that cytokine production as examined by ELISA paralleled the production of IL10 and TNF-α observed by flow cytometry. Also, upon gp120 stimulation, CCL2 was produced by PBMC (8,380 pg/ml) and by enriched monocytes (26,951 pg/ml), (Fig 1C). The induction of IL-10 and CCL2 observed in PBMC cultures was even higher when only monocytes were in the culture. In contrast, TNF-α detection was more elevated in PBMC cultures than in monocyte cultures (Fig 1C).

### 3.2. Targeted mutations in recombinant gp120 variants differentially affect CD4 and CCR5 co-receptor binding

Since it is well known that gp120 has nanomolar affinities for its CD4 receptor [22], we next focused on the gp120/CD4 interaction to determine whether the pro-inflammatory response to gp120 was induced by CD4 signaling or its co-receptor CCR5. We generated a panel of recombinant gp120 proteins with altered properties with regard to CD4 and co-receptor binding. Since a tripeptide located in the V1/V2 domain of the gp120 was previously shown to be involved in α₄β₇ binding [30] and thought to affect B cell proliferation [31], we evaluated the contribution of the variable regions in the cell receptor interaction and gp120 cytokine burst. The D368R change has been reported to affect CD4 binding [27, 28, 32], and the mutation R419D has been shown to affect binding to the CCR5 co-receptor [33]. Removal of the V1V2V3 and V5 variable regions was shown to favor the spontaneous sampling of the CD4-bound conformation [23, 24]. A detailed description of each recombinant gp120 variant is presented in Table 2. As previously shown [34, 35], the D368R change decreased the interaction of gp120 with CD4 while deletion of the V1V2V3V5 variable regions enhanced the interaction with CD4 and 17b, an antibody which preferentially recognizes the CD4-bound conformation. Of note, deletion of the V1V2V3V5 loops resulted in decreased CCR5 binding due to the absence of the V3 loop required for CCR5 engagement. The introduction of D368R into the dV1V2V3V5 mutant abrogated CD4 binding. As expected, the R419D mutation did not alter CD4 binding but significantly decreased the interaction with CCR5 and its surrogate, the 17b antibody. Interestingly, the R419D-induced decrease in 17b binding was only observed in the full-length gp120 but not in the dV1V2V3V5 context whereas it affected CCR5 binding in both contexts, indicating that while 17b is a good surrogate of co-receptor binding and recapitulates several binding properties of CCR5 some differences between this antibody and the co-receptor remain.

**Table 2:** Characterization of ligand binding to selected HIV-1_YU2_ gp120 proteins by immunoprecipitation.

gp120	CD4-Ig	17b	R5 (-sCD4)	R5 (+sCD4 10 μg/ml)^a^
**Wild type**	1	1	1	1 ^b^
**D368R**	0.023***	1.233	0.906	0.771
**R419D**	0.917	0.218**	0.064	0.097**
**ΔV1V2V3V5**	1.450	2.058^p = 0.0745^	0.373	0.224*
**ΔV1V2V3V5 D368R**	0.058***	2.529*	0.437	0.169***
**ΔV1V2V3V5 R419D**	1.469	2.517	0.110	0.069***
**ΔV1V2V3V5 R419D D368R**	0.039***	2.890	0.288	0.137**

*p = 0.05,

** p = 0.01,

***p = 0.001

^**a**^ CCR5 binding in presence of sCD4 was 3 folds higher than in its absence.

^**b**^ Normalization to 1 for wt, R5 interaction is 3 folds higher in presence of sCD4.

Statistical test for all samples is a paired t-test. Presentation of the different gp120 construct tested in the study and their ligation capacity with CD4-Ig, 17b antibody, CCR5 (-/+ soluble CD4). After migration of the immuno co-precipitate, quantification of the gel bands are expressed as relative to the wild type set at the value of 1.

### 3.3. Cytokine-inducing signaling by gp120 in monocytes is dependent on CD4

**Fig 2:**
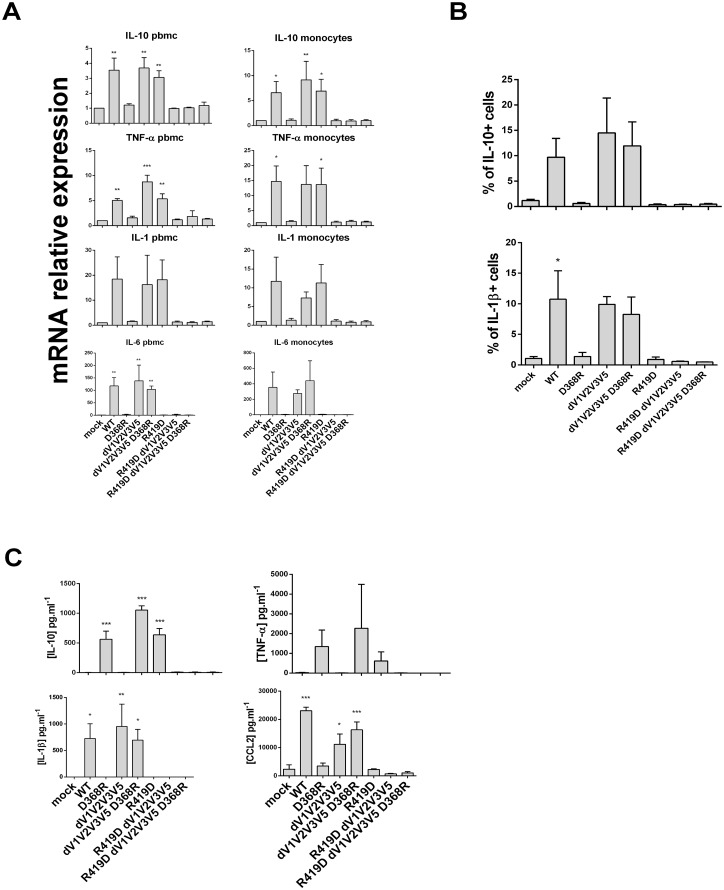
D368R and R419D mutations are deleterious for the cytokine burst but dV1V2V3V5 mutation can rescue D368R. Cytokines detection after cell stimulation with the gp120 constructs collection. A) mRNA expression of cytokines done by RT-qPCR, level of expression after stimulation with gp120 mutants is relative to the mock control set at 1 for each graph. Left panel: PBMC and right panel: monocytes. B) Flow cytometry analysis of PBMC CD14+ population, expression of IL-10 and IL1-ß are detected by intra-cellular staining after gp120 mutants’ stimulation. C) PBMC supernatant analysis by ELISA detection of IL-10, TNF-α, IL-1ß and CCL2 after stimulation with gp120 mutants. Statistic analysis were performed with variance test ANOVA and then with a t-tests to compare positive conditions to the mock control (* p<0.05 and ** p<0.01).

**Fig 3:**
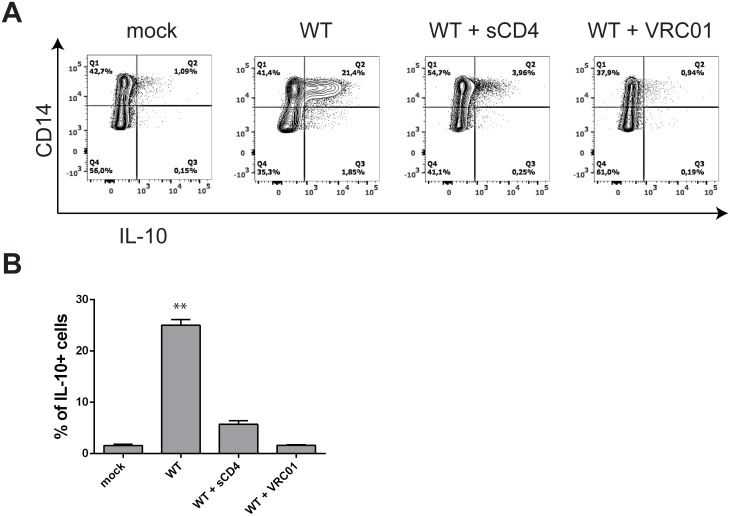
Blockade of gp120 CD4-binding site inhibits IL-10 production by monocytes. A) Representative experiment and B) flow cytometry analysis of IL-10 expression on PBMC CD3- CD14+ cells detected by intracellular staining after gp120 stimulation in absence or presence of sCD4 or the CD4-binding site VRC01 antibody at a molar ratio of 1:1 with gp120. Statistical analysis were performed with t-tests to compare to the mock control (** p<0.01).

We next sought to test how the different mutants affected the cytokine production elicited by WT gp120 that we observed in Fig 1. Examining a mean of 5 donors, we tested whether or not the different mutants were able to induce cytokine production, using three different approaches: RT-qPCR (Fig 2A), flow cytometry (Fig 2B) and ELISA (Fig 2C). We performed the assays with PBMC and monocytes isolated by negative selection. We observed a complete loss of cytokine production with the D368R mutation that abrogates gp120 binding to CD4, underlining that the absence of CD4 ligation is deleterious for gp120 signaling. This loss of immune modulation was observed both at the mRNA (Fig 2A) and protein (Fig 2B and 2C) levels. To further confirm the involvement of the CD4-binding site of gp120 on cytokine production, we stimulated PBMCs from a healthy donor with gp120 alone or in complex with soluble CD4 (sCD4) or the CD4-binding site VRC01 antibody. As shown in Fig 3, while gp120 alone was sufficient to induce the production of IL-10 in CD3- CD14+ cells, both sCD4 and VRC01 dramatically decreased IL-10 production. Thus, confirming the results obtained with the full-length D368R gp120 variant (Fig 2) indicating that CD4 binding is required for gp120-induced monocyte cytokine induction.

### 3.4. CCR5 co-receptor binding site contribution to cytokine signaling

Compared to WT gp120, the dV1V2V3V5 mutation induced the same amounts of IL-10 and pro-inflammatory cytokines such as TNF, IL-1 and IL-6. This mutated recombinant protein was able to bypass the D368R mutation as the combination of the two mutations D368R/dV1V2V3V5 led to cytokine production. Finally, anytime the R419D was present in any recombinant gp120 protein no induction of cytokine was observed. Our findings indicated that the interaction of gp120 with CD4 is mandatory to induce the cytokine burst, but this can be by-passed by an engineered recombinant gp120 protein spontaneously exposing the co-receptor binding site. Nevertheless, while the introduction of the R419D mutation within the dV1V2V3V5 context did not change the dV1V2V3V5 construct binding properties to specific ligands (Table 2) the cytokine production was drastically reduced (Fig 2).

While our results suggested that CCR5 co-receptor binding is important for gp120-induction of cytokines, maraviroc, a gp120-CCR5 interaction inhibitor, did not interfere with the production of cytokines (data not shown). This finding underlies the necessity to further characterize the mechanisms involved in gp120 signaling.

### 3.5 Role of gp120-CD4 interaction on chemo-kinesis of CD4+ T cells and monocytes

**Fig 4:**
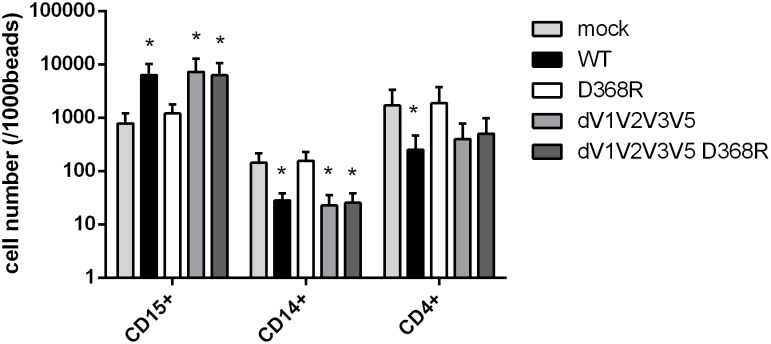
gp120 induces neutrophils and lymphocytes migration but inhibits monocytes migration. Transwell immune cells migration analyzed with evaluation of PBMC subpopulation counts by flow cytometry. 2*10^5 cells were incubated with gp120 mutants and then cultured in insert with a 3μm membrane. Active migration of cells was evaluated after harvest of the medium in the wells. Cells were stained with anti CD15, CD14 and CD4 antibodies and counts were normalized to 1000 beads. Statistic analysis were performed with variance test ANOVA and then with a t-tests to compare positive conditions to the mock control (* p<0.05 and ** p<0.01).

The importance of selective cell chemotaxis during HIV infection is an important subject of investigation. The mechanism of this recruitment may be direct attraction of target T cell population via viral products or alternatively, via the action of other immune cells [11, 36–38]. Here we evaluated whether our panel of recombinant gp120 variants was able to induce cell chemokinesis (Fig 4). Immune cell attraction by chemokinesis has been shown to expand mucosal and systemic inflammation in the context of HIV-1 infection [9, 10]. In accordance with the previous results showing no cytokine production upon R419D mutant stimulation, R419D did not induce any PBMC chemokinesis (data not shown). Thus, we focused on the other gp120 mutants. The chemo-kinesis test reproduced the results obtained with the cytokine burst: gp120 WT and dV1V2V3V5 enhanced the chemo-kinesis of neutrophils while the D368R mutation abrogated this effect with results close to the mock control in the full-length context but not within the variable regions (dV1V2V3V5) deleted recombinant gp120 protein (Fig 4). On the other hand, monocytes were negatively impacted by the WT recombinant gp120 with a reduction of chemo-kinesis and the D368R construct had no impact on monocytes kinesis.

## 4. Discussion

HIV-1 enters the host cells by engaging two receptors, CD4 and one G protein coupled chemokine receptor, CCR5 or CXCR4. In this study, we focused on the importance of these receptors in the HIV envelope protein gp120 induction of cytokines. Using PBMCs from healthy donors, we identified a strong and diverse cytokine burst in response to gp120 mainly due to monocytes. Using different approaches, we identified monocytes as significant producers of IL-10, IL-1, IL-6 and CCL2 upon gp120 stimulation. These results were in accordance with previous work reporting cytokine production in the context of HIV infection [14, 39]. This burst includes IL-6, a cytokine necessary in gp120 suppressive induction as previously reported [40] but also IL-10 [6, 41]. Although monocytes were the primary responding cells, we cannot exclude the contribution of other cell populations. For example, T cells produce a lot less IL-10 than monocytes, even though IL-10 mRNA is up-regulated in T cells [6]. Our results point out to an essential role of monocytes in HIV pathogenesis and support the need of further investigation on HIV gp120 protein interaction with monocytes.

Production of chemokines has also been reported as a signature of inflammation [11, 12, 42]. The mechanisms of chemokine burst in the context of HIV infection have not been thoroughly assessed. Here we decided to use engineered recombinant gp120 to evaluate different regions in gp120 in the induction of IL-1, IL-6, IL-10, TNFα or CCL20 cytokines. Importantly, our data show that decreasing CD4 interaction by introducing the D368R mutation in gp120 abrogates the cytokine burst. Thus, CD4 is a first necessary step in the induction of the cytokine burst. Of note, despite that the CD4 binding site is highly conserved among different HIV-1 isolates, we cannot rule out that differences might be observed using gp120 from additional strains.

Interestingly, the phenotype of the D368R mutant (i.e., no cytokine induction) was reversed by the loops deletion. This observation suggests that the spontaneous sampling of the CD4-bound conformation upon the loops deletion is sufficient to mediate gp120 signalling.

Both innate and adaptive immune responses against a pathogen such as the HIV require selective migration of immune cells. We developed a chemokinesis assay on whole blood in order to investigate the impact of gp120 on each population of the circulating immune cells. It has been documented that HIV impacts T cell migration [36, 38] but little is known of its effect on other cell types. We observed neutrophil chemokinesis upon gp120 stimulation. HIV is already known to stimulate granulocytes through the gp120 immunoglobulin superantigen binding site [9] but it is still unclear whether neutrophils express CD4 on their cell surface [43]. Neutrophil migration can be stimulated through formyl peptide receptor (FPR) but whether FPR interact with gp120 or not still has to be investigated. Of note, experiments with purified populations of granulocytes or monocytes showed the same results (data not shown) that is positive migration of neutrophils and reduced migration of monocytes. Again, our results underline the potential role of non-T cell types like monocytes and neutrophils in gp120-induced pathogenesis.

Immune hyperactivation status is a crucial feature of the pathogenesis of HIV infection. This chronic inflammation is improved, but not fully corrected by ART. Notably, higher levels of immune activation in virally suppressed individuals are associated with a number of non-AIDS defining clinical complications [2]. Here, we clarify the features of gp120 that modulate the cytokine burst in monocytes, and complement previous work by characterizing the profile of this functional response. While this mechanism may significantly contribute to immune dysregulation in untreated progressive HIV disease, it will be important to define whether the residual gp120 antigen load that can be present in individuals on ART [14] is sufficient to elicit stimulation of the myeloid compartment. If so, strategies aiming to maximize gp120 protein or residual virus clearance (such as antibody-based approaches) could be beneficial as adjuvant therapy for patients who are poor immunological responders on ART.
